# Numerical Modelling of a Distributed Acoustic Sensor Based on Ultra-Low Loss-Enhanced Backscattering Fibers

**DOI:** 10.3390/s21206869

**Published:** 2021-10-16

**Authors:** Lieke Dorine van Putten, Ali Masoudi, James Snook, Gilberto Brambilla

**Affiliations:** Optoelectronic Research Centre, University of Southampton, Southampton SO17 1BJ, UK; ldvp1v18@soton.ac.uk (L.D.v.P.); j.h.snook@soton.ac.uk (J.S.); gb2@orc.soton.ac.uk (G.B.)

**Keywords:** enhanced backscattering fibers, DAS, DVS, phase-OTDR, numerical modelling, distributed acoustic sensor, ultra-low loss

## Abstract

In this study, a distributed acoustic sensor (DAS) was numerically modeled based on the non-ideal optical components with their noises and imperfections. This model is used to compare the response of DAS systems to standard single-mode fibers and ultra-low loss-enhanced backscattering (ULEB) fibers, a fiber with an array of high reflective points equally spaced along its length. It is shown that using ULEB fibers with highly reflective points improves the signal-to-noise ratio and linearity of the measurement, compared with the measurement based on standard single-mode fibers.

## 1. Introduction

Distributed optical fiber acoustic sensors (DASs), also known as distributed vibration sensors (DVSs), have found a wide range of applications in areas that require vibration and tremor (e.g., earthquakes) monitoring along extended structures such as railways, highways, sub-sea cables, and pipelines [1,2,3,4,5,6,7,8,9].

The majority of DAS systems utilize phase-sensitive optical time-domain reflectometry (ϕ-OTDR) to measure strain along a sensing fiber [10]. In this technique, variations at any given section of the sensing fiber are measured by analyzing the phase difference between the backscattered light from two ends of that section. Strain analysis based on the phase of the backscattered Rayleigh light has two main drawbacks: (a) the signal decay along the sensing fiber, which results in deterioration of signal-to-noise ratio (SNR) towards the far-end of the fiber and, (b) measurement error due to the non-linear changes in the phase of the backscattered light that is associated with the redistribution of inhomogeneities within the fiber [11]. Since the length of the fiber is of vital importance for the vast majority of applications, researchers have focused on developing methods to increase the sensing range of DAS systems using different techniques. Some of these techniques are based on advanced signal processing, such as utilizing machine learning algorithms to denoise the signal. Others are based on the amplification of the signal by remotely pumped Erbium-doped fiber amplifiers (EDFA), Raman inline amplifiers, or a combination of the two [12,13,14,15]. As for suppressing the effect of the non-linear changes in the backscattered phase, different approaches have been proposed. Some of these approaches use complex high-speed modulators and detectors to measure multiple uncorrelated Rayleigh backscattering to reduce the non-linear effect [16,17], while others use chirped probe pulse and cross-correlation of coherent Rayleigh noise (CRN) to eliminate the need for backscattering phase analysis altogether [18]. In addition, some studies demonstrated the link between the pulse duration and the non-linear phase term, showing how reducing the width of the probe pulse can reduce the effect of the non-linear term [19]. However, these techniques rely either on complex data analysis techniques or a trade-off between measurement precision and SNR.

A different technique that has been utilized to increase the performance of DAS is ultra-weak fiber Bragg gratings (UWFBG). First reported in 2017, UWFBG written in the sensing fiber has shown an improvement in the SNR of DAS systems. In 2020, Li et al. demonstrated an increase of 21.1 dB in SNR whilst accurately measuring vibration and temperature, simultaneously [20,21,22,23]. However, performance enhancement based on UWFBG comes at the cost of higher signal attenuation. As a result, UWFBG fibers are best reserved for applications with a relatively short range or for extending the range of conventional DAS systems by a few tens of kilometers [23,24].

In 2019, Hicke et al. presented a novel approach to reduce the non-linear phase response using a series of localized point reflectors [25,26]. More recently, Masoudi et al. have demonstrated a long-range DAS system using an ultra low-loss enhanced-backscattering (ULEB) fiber that is also based on point reflectors [27]. ULEB fibers use point reflectors to enhance the back-reflected light, but the inscription of the reflectors is well controlled to avoid excess losses. The optical attenuation of the ULEB fiber in ref. [27], for instance, was measured to be 2.05 dB/km, which translates to an excess loss of 0.05 dB/km over a standard telecom fiber. The low attenuation level of ULEB fibers makes them an ideal solution for applications that require high sensitivity over a long sensing range.

In addition to the enhancement of the backscattered signal, ULEB fibers also improve the precision of the strain measurement. Conventionally, DAS systems rely on the phase of the Rayleigh backscattered radiation from the randomly distributed inhomogeneities in the fiber to measure vibrations. It has been shown that the phase unwrapping procedure based on the phase of the Rayleigh backscattered light has a linear term, which corresponds to the elongation of the fiber, Δl, and a non-linear term associated with the redistribution of inhomogeneities in the fiber [19]:

(1) $$\Delta \Phi =0.78\times \frac{4\pi n}{\lambda }\Delta l+\Delta \varphi$$

where λ is the wavelength of the probe light, *n* is the refractive index of the fiber, and Δφ is the non-linear phase term. Since the value of the Δφ depends on the relative position of the inhomogeneities in the fiber, any changes in the fiber that result in redistribution of these inhomogeneities is manifested as error in the measurement. In ULEB fibers, the non-linear phase term is eliminated by introducing point reflectors with a reflectivity far in excess of Rayleigh backscattered light. By using point reflectors, the phase of the reflected light for any given section of the fiber only depends on a single reflector and will be independent of the interaction between the backscattered light from hundreds of scattering centres. Therefore, the non-linear phase term is eliminated from the phase-difference calculation [27]:

(2) $$\Delta \Phi =0.78\times \frac{4\pi n}{\lambda }\Delta l$$

This leads to a reduced signal degradation and higher measurement precision without the need for expensive high-speed modulators or complex signal processing.

To design and develop DAS systems with a higher SNR and better linearity, it is of great importance to understand the noise contribution of each part of the system. In 2017, Masoudi et al. numerically analyzed the behaviour of a phase-sensitive DAS [11] by numerically modeling each part of the DAS system separately, and combining them to simulate the entire system. The numerical model developed, however, was for an ideal system with ideal components. In this study, a more comprehensive model of DAS, which includes laser phase noise, digitizer quantization, and detector and optical amplifier noises is presented. The model is subsequently used to numerically model the effect of ULEB fibers on the performance of DAS systems.

## 2. Theory

To numerically model the backscattered light in a ϕ-OTDR system, the interaction between different elements within the system needs to be simulated. The Rayleigh backscattered light can be analyzed by modelling the interaction between the probe pulse and the inhomogeneities in the fiber. To model this, the original model used six modules:

- A module to model the sensing fiber: This module models the inhomogeneities in the fiber. It generates a two-column array with one column determining the locations of the inhomogeneities along the fiber with the other column determining their sizes, thus the intensity of the scattering from the corresponding location.
- A module to simulate the probe pulse created by the laser: This module creates a m×n matrix where *m* represents the spatial shape of the probe pulse while *n* represents the frequency components within it.
- A module to simulate the effect of strain on the scattering in the fiber: This module simulates the effect of induced strain on one or multiple locations on the fiber by rearranging the position of the scattering centres within the fiber.
- A module to calculate the backscattered electric field: This module calculates the backscattered electric field for each section of the fiber by evaluating the convolution sum of the matrices that represent the fiber and probe pulse.
- A module to analyze the effect of the imbalanced Mach-Zehnder interferometer (IMZI): This module delays the backscattered electric field from the previous module and adds it to itself. The output of the IMZI module represents the electric fields received by the three detectors.
- A module to calculate the response of the photodetectors: This module converts the electric field to intensity, measured at the photodetector, by integrating the power of the electric field over a certain time interval, which is determined by the bandwidth of the detector.

**Figure 1:**
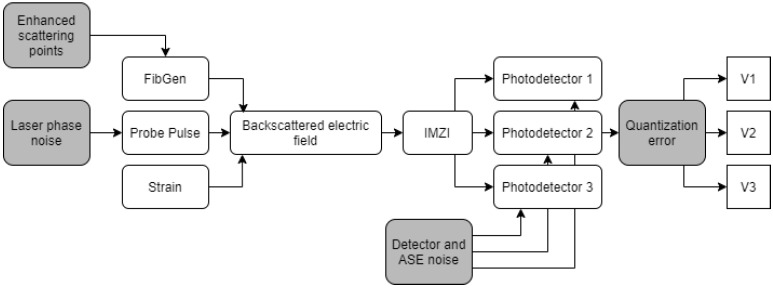
Block diagram showing the modules used to simulate the output of an ideal DAS system (white) as well as the modules used to imitate various noises in the system (grey).

To provide a more realistic behavior of the system, four different sources of noise and errors are added to the model: (1) laser phase noise, (2) detector noise, (3) optical amplifier noise, and (4) digitizer quantization error. A block diagram showing these modules and their corresponding noises is shown in Figure 1.

### 2.1. Laser Phase Noise

Fluctuations in the frequency and amplitude of a laser are manifested as noise at the laser output. Laser phase noise, in particular, is associate with a drift in the frequency of a laser. This noise results in a small drift in the frequency and hence, the wavelength of the seed laser between each pulse. In an ideal system, the frequency output of the laser is fixed. In reality, however, small variations in the frequency are present due to changes in the temperature and current of the laser, especially over longer time spans [28]. The magnitude of these variations depends largely on the quality of the laser and its driver. A high-quality narrow linewidth laser can exhibit a frequency noise as low as 30 Hz/$\sqrt{Hz}$ over the 100 μs time span. For less expensive lasers, the phase noise could be orders of magnitude higher. In this study, the laser phase noise was assumed to have a wavelength drift of 2.5 pm per second. Realistically, the frequency of the laser has a stochastic behaviour and could randomly increase or decrease. However, over a short time frame (e.g., one second), it can be assumed that the laser frequency is moving in one direction in order to simulate the effect of 1/f noise. Such frequency drift was modelled by a continuous change in the frequency of the laser in the upward or downward direction. This condition was simulated by always adding a random percentage of the maximum phase noise to the wavelength of the laser for each new pulse. To simplify the simulation, the frequency drift was assumed to be negligible for the duration of the probe pulse.

### 2.2. EDFA and Detector Noises

The detector noise is added to the photodetector module. The detector noise is calculated based on the noise-equivalent power (NEP) of a typical amplified photodetector (2.5 pW/$\sqrt{Hz}$) with an electrical bandwidth of 100 MHz.

In addition, an optical pre-amplifier was incorporated into the new model. The addition of this amplifier boosts the backscattered signal, but also adds noise to the system. Amplified spontaneous emission (ASE) noise from EDFA has two components: (a) ASE-ASE beat noise and (b) signal-ASE beat noise. ASE-ASE beat noise is formed due to the interaction of the broad-band ASE with itself. This beating appears as a noise on the detector with an NEP of [29]:

(3) $$P_{ASE-ASE}=\sqrt{4S_{ASE}^{2}\;B_{opt}\;B_{elec}}$$

where $S_{ASE}$ is the ASE power per unit bandwidth and $B_{opt}$ and $B_{elec}$ are the optical and electrical bandwidths of the system, respectively. Signal-ASE beat noise is formed due to the interaction between the signal and the ASE. The NEP of this noise is given by [29]:

(4) $$P_{SIG-ASE}=\sqrt{4GP_{S}\;S_{ASE}\;B_{elec}}$$

where *G* is the gain of the EDFA and $P_{S}$ represents the power of the backscattered light. The ASE and detector noises are added to the amplified backscattered signal to simulate the signal before digitization.

### 2.3. Quantization Error

The quantization error is also included in the detector module to simulate the behaviour of the data acquisition system. Digital signal processing requires discrete data that is generated from an analog to digital converter (ADC). ADCs convert analog data to discrete voltage levels. The simulated intensity at the output of the photodetector module was converted from an analog input into digital values by a 12-bit digitizer, a digitization resolution used in conventional data acquisition cards.

### 2.4. Scattering Points

**Figure 2:**
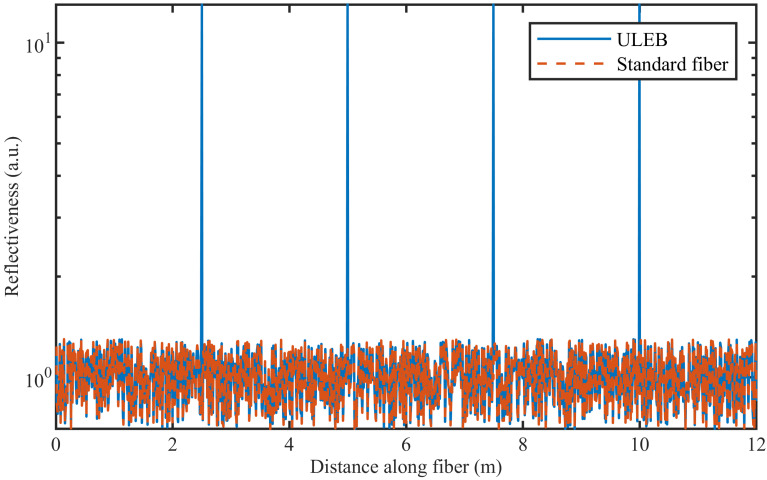
Example of scatter points along a standard fiber and along a ULEB fiber.

The last addition made is to the module that simulates the fiber. Previously, the scattering points with similar scattering levels were used to simulate inhomogeneities in the fiber. To create evenly spaced high reflective points, the module was modified to allow for the introduction of high reflective points with a well-defined spacing. In Figure 2, an example of the scattering points along the fiber is shown for a fiber with and without reflectors.

## 3. Numerical Modelling Procedure

For this simulation, the sensing system was assumed to have a 10 km fiber, of which the Rayleigh backscattered light from the last 20 m was simulated. This corresponds to a round-trip loss of 4 dB. In addition, it was assumed that the sensing system has an EDFA with a 19 dB gain to amplify the backscattered light. The probe pulse used in the model was assumed to have a duration and peak power of 10 ns and 200 mW, respectively. The peak power of the probe pulse was set to 200 mW to avoid any nonlinear effects in the fiber. For such a probe pulse, the power of the Rayleigh backscattered light that reaches the DAS system from the far end of the fiber would be 6.3 nW. The Rayleigh backscattered light is then boosted to 500 nW by the EDFA and combined with the noises from the detector and EDFA to simulate the signal before digitization. The noise floor of a detector with an NEP of 2.5 pW/$\sqrt{Hz}$ and 100 MHz bandwidth would be 30 nW. The noise from the EDFA can be calculated using Equations (3) and (4). Using a narrow bandwidth FBG filter with a 10 GHz bandwidth limits the optical bandwidth of the system to 0.08 nm. Hence, the ASE-ASE and ASE-signal beat noises for the EDFA with $S_{ASE}$ = 2 μW/nm would be 32 nW and 56 nW, respectively. Hence, the combined NEP of the detector and EDFA would be 113 nW, which corresponds to an SNR of 4.5. The path imbalance of the IMZI was set to 2 m to match the round-trip time of the probe pulse.

For the first test, ideal and a non-ideal DASs were simulated to evaluate the effects of the laser, detector, and EDFA noises on the output of the system. For this simulation, a 1150 Hz sinusoidal strain with a peak-to-peak amplitude of 1 μϵ between the 8th and 9th meter was used.

After the initial noise analysis, the SNR of standard and ULEB fibers are compared. For this analysis, the reflectivity of the ULEB fiber is set to 100 times higher than the maximum Rayleigh backscattering level. To ensure consistency, the same sinusoidal strain is imposed on both fibers. In addition, to study the precision of the strain measurement between two fibers, the time-domain results obtained from the two fibers are juxtaposed.

To examine the effect of strain on the ULEB fiber and to ensure that the enhanced fiber has a linear strain response, four different strain levels from 0.25 μϵ to 1 μϵ were applied at a fixed frequency and location on both the standard and ULEB fibers. In addition, the results from this simulation were used to compare the accuracy and precision in the measurements from ULEB and standard fibers.

Next, the effect of the reflectivity of the enhanced points on the noise level of the system was studied. The strain level and the setup are kept unchanged and only the reflectivity of the enhanced scattering points are varied from the Rayleigh level (no enhancement) to 250 times the Rayleigh backscattering level. The noise levels in these fibers were compared to analyze the relationship between the reflectivity of the point reflectors and the SNR of the system.

Finally, the response of the DAS for a scenario where the strain is imposed on the section of the fiber with an enhanced scattering point was studied. So far, such an arrangement has been avoided during experiments, but in real life applications, this cannot be guaranteed. This simulation was carried out to assess how well the data analysis performs when the strain is imposed on a section of the fibre with a reflector. The ADC sampling rate in all tests was set at 5 GSa/s.

## 4. Results

**Figure 3:**
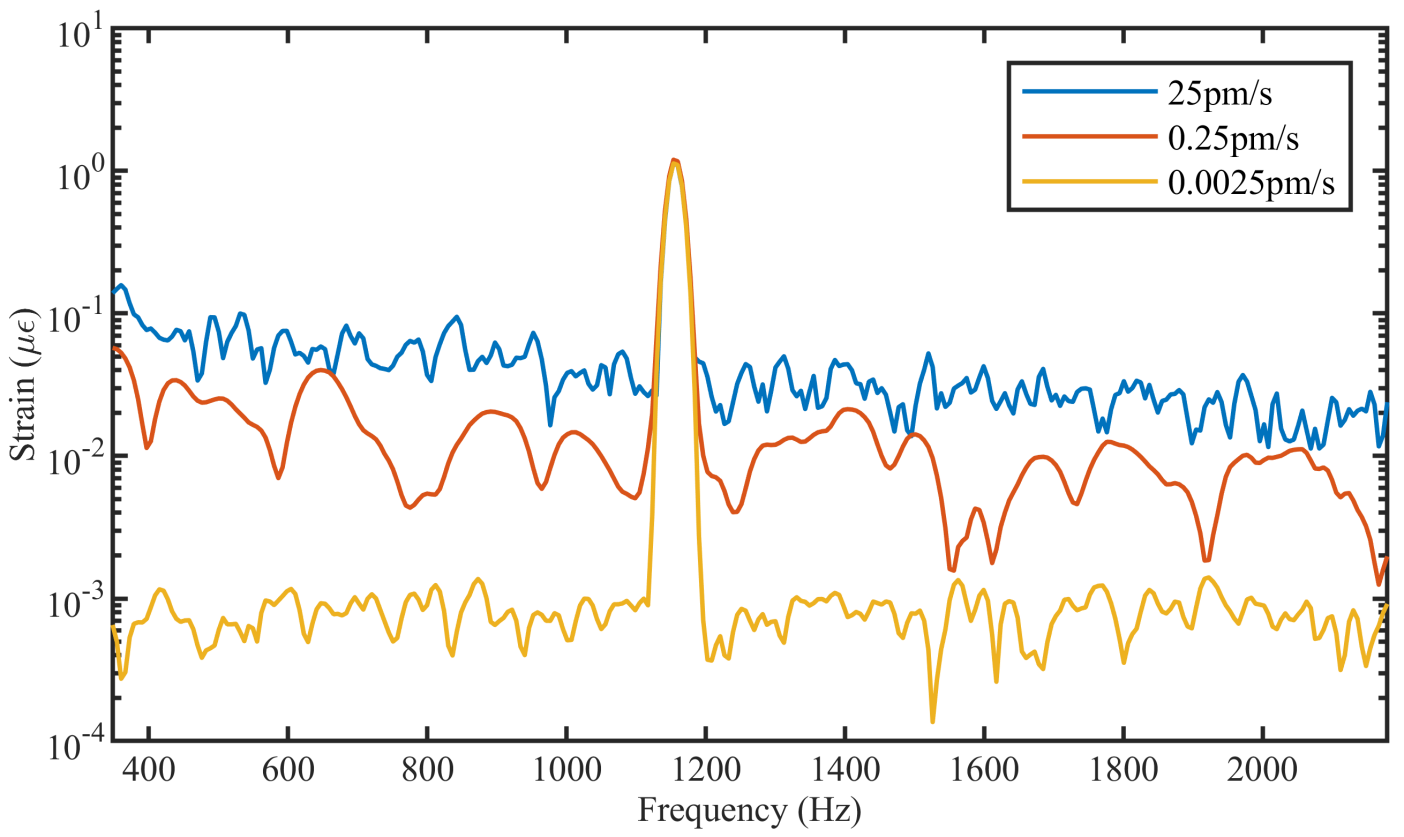
FFT of signal measured in 3 different systems with different levels of laser phase noise.

The first simulation was carried out to evaluate the effect of laser phase noise on the performance of the DAS numerical model. In this simulation, the system response to a laser with three different phase noise levels was assessed (Figure 3). From this figure, it can be seen that the laser with a broader linewidth (i.e., the laser with larger frequency drift) exhibits a higher noise floor (blue trace) compared with the more stable laser with lower frequency drift (yellow trace). This indicates that the phase noise added to the laser module is working as expected. The effect of EDFA and detector noises were not added since the aim of the first simulation was only to analyze the effect of laser phase noise on the DAS system.

**Figure 4:**
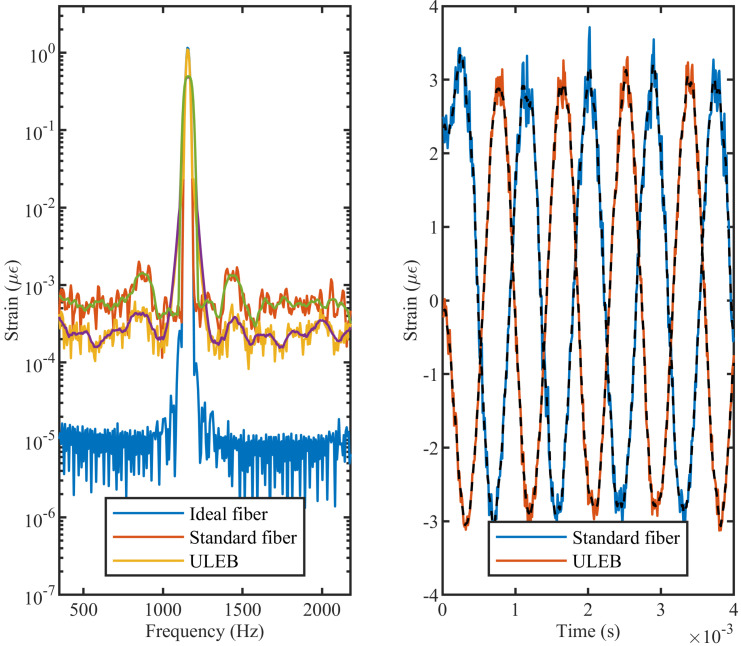
(**left**) FFT of signal in an ideal scenario, a standard fiber, and a ULEB, showing the difference in SNR. (**right**) Time-domain signal showing the difference in accuracy between measurements in a standard fiber and a ULEB.

In Figure 4, the frequency spectra of dynamic strain for three different fibers are shown. The three fibers are: (a) a standard telecom fiber interrogated by a system with no noise (blue trace), (b) a standard telecom fiber interrogated by a system with noise (red trace), and (c) a ULEB fiber with ×100 reflectors, interrogated by a system with noise (yellow trace). The vibration at 1150 Hz shows the same peak for all three fibers. From this diagram, it can be seen that the average noise floor of the ULEB fiber spectrum is 9 dB lower than that of the standard telecom, which is in agreement with the experimental results from [26]. The lower noise floor is achieved only by changing the properties of the fiber from standard to ULEB. From the time domain data shown in Figure 4 right, it can be seen that the ULEB fiber yields a strain measurement with higher precision and lower noise.

**Figure 5:**
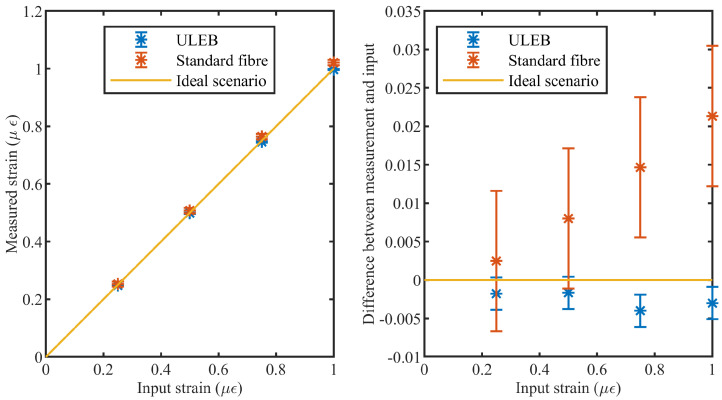
(**left**) Measured strain compared to input strain. (**right**) Absolute difference between the input strain and measurements.

To confirm that a DAS system using a ULEB fiber exhibits higher strain precision, the model was tested by simulating different strain levels on both the ULEB and standard fibers. The results are show in Figure 5. It can be seen that both fibers have a high degree of linearity. However, the simulation result of the system based on the ULEB fiber shows a significant improvement in strain precision, reducing the standard deviation by over a third (from 1.67% to 0.51%). This observation agrees with the theoretical analysis provided in Equations (1) and (2). According to Figure 5, the ULEB fibre shows a lower strain measurement error, which can be associated with the lower SNR level of this type of fiber. These results are in agreement with the previous experimental observations [16,27].

**Figure 6:**
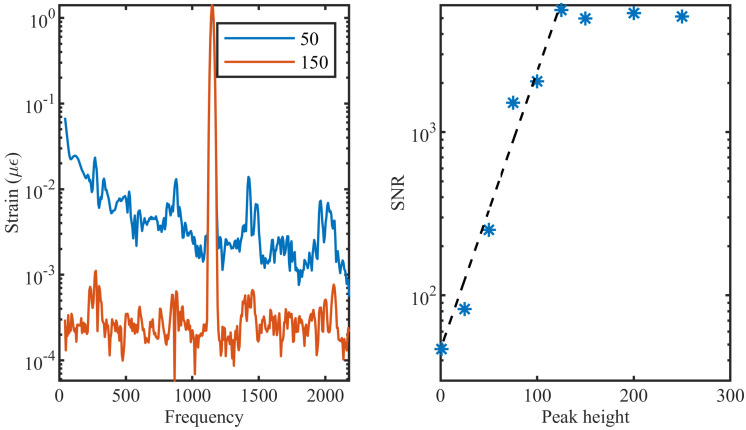
(**left**) FFT of the signal for two different ULEB fibers, one with a maximum reflectivity of 50 and the other with 150. (**right**) SNR as a function of different reflectivity levels.

To analyze the effect of the reflection level of the point reflectors in the ULEB fiber on the noise level of the system, six different levels of reflectivity were modeled, varying from no enhanced reflectivity (i.e., Rayleigh level) to 250× reflectivity. For these analyses, the detector and EDFA noises were not added since the randomness in these noises could have had a higher influence on the SNR than the reflectivity level itself. The result of this simulation can be seen in Figure 6. Reflectors with higher peak reflectivity exhibit a lower noise floor, and thus a better SNR. However, it can be seen that there is no further improvement in SNR for reflectivities higher than 100×. This behaviour can be associated with the relative value of the signal level to the noise floor and the digitization resolution of the ADC. For reflectors with reflectivity above 100, the SNR level drops below the digitization level of the ADC and, consequently, any further improvement in the reflectivity will not be registered. This response will change if the noise floor of the system increases or if a higher resolution ADC is used.

**Figure 7:**
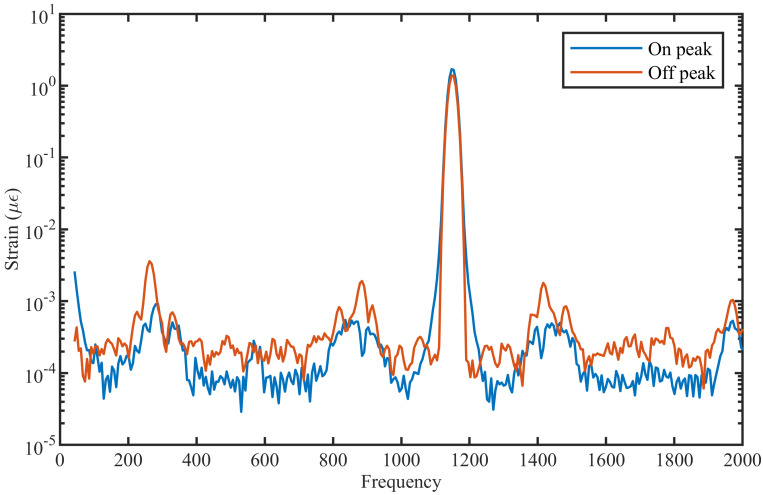
FFT of the signal when the vibration is on and off the enhanced reflectivity peak.

For the last analysis, an 1150 Hz dynamic strain with amplitude of 0.5 μϵ was applied at two different locations of the ULEB fiber; one between two enhanced reflective points and the other overlapping with a reflector. For this simulation, a ULEB fiber with an enhanced reflectivity of 75× above the Rayleigh backscattering level was used. From Figure 7, it can be seen that a DAS system can spatially resolve dynamic strain at each point along the ULEB fiber with the same SNR. This outcome shows that ULEB fibers can be used in a wide range of DAS applications to improve the SNR with no limitation on the position of the reflectors.

## 5. Conclusions

The simulation results shown in this paper give an insight into the behaviour of a realistic DAS system through the addition of noises and imperfections into the ideal model. The effect of the noise levels and different fiber types on the behavior of the DAS system were studied. The results showed that ULEB fibers have the potential to improve the SNR of the DAS system by nearly one order of magnitude, without the need for costly components or advanced signal processing techniques. It was shown that the accuracy of the frequency and strain measurement is not affected by using ULEB fibers, and that the DAS system showed an improvement in the precision of strain measurements using this type of fiber. The strain measurement based on the ULEB fiber showed an improvement in the measurement precision by a factor of three. In the time-domain data, the reduction in noise level was also visible.

This model provides a useful tool to study how noises and imperfections inherent in the optical components that form a DAS system can influence its performance. The model can be used to evaluate the effectiveness of novel techniques employed to reduce the noises of DAS systems, such as by using different types of speciality fibers, or by using frequency diversity. Optimising a DAS system through numerical modelling before performing lab experiments can save time and is significantly more cost effective. Furthermore, this study shows how ULEB fibers can be effectively used to increase the SNR. It also demonstrates that these fiber types can work effectively when the vibration is directly on one of the enhanced reflectivity points.

## Data Availability

Data underlying the results presented in this paper are available at https://doi.org/10.5258/SOTON/D1883, accessed on 10 May 2021.
